# Tris[2-(deuteriomethyl­sulfan­yl)­phen­yl]­phosphine deuteriochloro­form 0.125-solvate

**DOI:** 10.1107/S1600536808010817

**Published:** 2008-04-23

**Authors:** Richard Chee Seng Wong, Mei Lee Ooi, Hidehiro Sakurai, Seik Weng Ng

**Affiliations:** a Department of Chemistry, University of Malaya, 50603 Kuala Lumpur, Malaysia; b Research Center for Molecular Nanoscience, Institute for Molecular Science, Myodaiji, Okazaki 444-8787, Japan

## Abstract

The title deuterated tripodal phosphine, C_21_H_12_D_9_PS_3_·0.125CDCl_3_, crystallizes as two independent mol­ecules, one of which lies on a general position and the other about a threefold rotation axis, and as a deuteriochloro­form solvate. The solvent mol­ecule is disordered about a site of symmetry 3, so that the ratio of phosphine to solvent is 8:1. The P atom adopts a pyramidal coordination geometry.

## Related literature

For the synthesis and crystal structure of tris­[(2-methyl­sulfan­yl)phen­yl]phosphine, see: Meek *et al.* (1976 ▶); Uttecht *et al.* (2005 ▶).
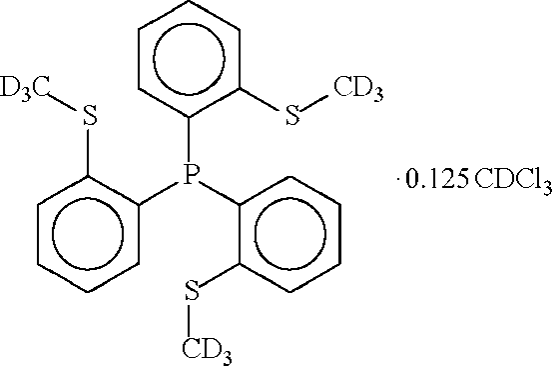


## Experimental

### 

#### Crystal data

C_21_H_12_D_9_PS_3_·0.125CDCl_3_
*M* *_r_* = 424.63
Hexagonal, 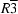

*a* = 23.090 (1) Å
*c* = 25.144 (1) Å
*V* = 11610 (1) Å^3^
*Z* = 24
Mo *K*α radiation
μ = 0.52 mm^−1^
*T* = 100 (2) K
0.20 × 0.15 × 0.10 mm

#### Data collection

Bruker SMART APEX diffractometer
Absorption correction: multi-scan (*SADABS*; Sheldrick, 1996 ▶) *T*_min_ = 0.925, *T*_max_ = 1.000 (expected range = 0.878–0.949)
36814 measured reflections
5929 independent reflections
4200 reflections with *I* > 2σ(*I*)
*R*_int_ = 0.089

#### Refinement

*R*[*F* ^2^ > 2σ(*F* ^2^)] = 0.062
*wR*(*F* ^2^) = 0.194
*S* = 1.18
5929 reflections
329 parameters
12 restraints
H-atom parameters constrained
Δρ_max_ = 1.14 e Å^−3^
Δρ_min_ = −0.49 e Å^−3^

### 

Data collection: *APEX2* (Bruker, 2007 ▶); cell refinement: *SAINT* (Bruker, 2007 ▶); data reduction: *SAINT*; program(s) used to solve structure: *SHELXS97* (Sheldrick, 2008 ▶); program(s) used to refine structure: *SHELXL97* (Sheldrick, 2008 ▶); molecular graphics: *X-SEED* (Barbour, 2001 ▶); software used to prepare material for publication: *publCIF* (Westrip, 2008 ▶).

## Supplementary Material

Crystal structure: contains datablocks global, I. DOI: 10.1107/S1600536808010817/tk2251sup1.cif

Structure factors: contains datablocks I. DOI: 10.1107/S1600536808010817/tk2251Isup2.hkl

Additional supplementary materials:  crystallographic information; 3D view; checkCIF report

## Figures and Tables

**Fig. 1.**
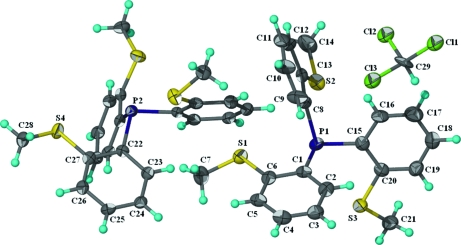
Thermal ellipsoid plot (Barbour, 2001) of the two independent (CD3S-2-C6H4)3P molecules showing atom labelling; the P2-molecule is located on a threefold axis of symmetry. Displacement ellipsoids are drawn at the 70% probability level. The disordered solvent molecule is omitted for clarity, and H and D atoms are shown as spheres of arbitrary radii.

## supplementary crystallographic information

### Comment

Tris[(2-methylsulfanyl)phenyl]phosphine is a tripodal ligand that yields a number of adducts with transition metals. The compound crystallizes without any solvent (Uttecht *et al.*, 2005). We intended to synthesize the deuterated title compound to examine their coordination patterns. The present deuteriochloroform solvate (Scheme I, Fig. 1) is isostructural with the reported solvent-free compound, whose crystal structure has been described in detail. The deuterated chloroform molecule is disordered, and appears to occupy a only small portion of the unit cell. Its presence is not sufficient to cause much change in the unit cell volume.

### Experimental

The procedure was adapted from a reported procedure (Meek *et al., *1976). *d_3_*-2-Bromothioanisole (2.50 g, 0.012 mol) was dissolved in dry ether (13 ml) at 273 K. To the solution was added over 2 h 1.6 *M n*-butyllithium in *n*-hexane (8 ml). A white precipitate separated after half the *n*-butyllithium was added. After the full quantity of the reagent was added, stirring was continued for another hour. Phosphorus trichloride (0.56 g, 0.004 mol) in ether (8 ml) was added over 3 h. The mixture was then allowed to warm up to room temperature before being hydrolyzed with 0.2 N hydrochloric acid (8 ml). The white solid was collected and washed with distilled water, ethanol and ether. The compound (1.216 g, 0.003 mol, 70% yield) was recrystallized from deuterated chloroform.

### Refinement

Carbon-bound H-atoms were placed in calculated positions (C—H 0.95 to 1.00 Å) and were included in the refinement in the riding model approximation, with *U*(H) set to 1.2 to 1.5*U*(C). Scattering factors used for deuterium were those of hydrogen.

The CDCl_3_ molecule was refined as a complete molecule of 1/8 occupancy about a site of symmetry, 3. The displacement factors of the three chloride atoms were restrained to be identical. The three C–Cl distances were restrained to within 0.01 Å of each other as were the Cl···Cl distances; the anisotropic temperature factors of the carbon atom were restrained to be nearly isotropic.

The final difference Fourier map had a peak 1 Å from P1 but was otherwise featureless.

### Crystal data

**Table:**

C_21_H_12_D_9_PS_3_·0.125CDCl_3_	*Z* = 24
*M**_r_* = 424.63	*F*_000_ = 5214
Hexagonal, *R*3	*D*_x_ = 1.458 Mg m^−^^3^
Hall symbol: -R 3	Mo *K*α radiation λ = 0.71073 Å
*a* = 23.090 (1) Å	Cell parameters from 2990 reflections
*b* = 23.090 Å	θ = 2.6–21.9º
*c* = 25.144 (1) Å	µ = 0.52 mm^−^^1^
α = 90º	*T* = 100 (2) K
β = 90º	Diamondoid, colorless
γ = 120º	0.20 × 0.15 × 0.10 mm
*V* = 11610 (1) Å^3^	

### Data collection

**Table:**

Bruker SMART APEX diffractometer	5929 independent reflections
Radiation source: fine-focus sealed tube	4200 reflections with *I* > 2σ(*I*)
Monochromator: graphite	*R*_int_ = 0.089
*T* = 100(2) K	θ_max_ = 27.5º
φ and ω scans	θ_min_ = 1.3º
Absorption correction: Multi-scan(SADABS; Sheldrick, 1996)	*h* = −29→29
*T*_min_ = 0.925, *T*_max_ = 1.000	*k* = −29→30
36814 measured reflections	*l* = −30→32

### Refinement

**Table:**

Refinement on *F*^2^	Secondary atom site location: difference Fourier map
Least-squares matrix: full	Hydrogen site location: inferred from neighbouring sites
*R*[*F*^2^ > 2σ(*F*^2^)] = 0.062	H-atom parameters constrained
*wR*(*F*^2^) = 0.194	*w* = 1/[σ^2^(*F*_o_^2^) + (0.1*P*)^2^] where *P* = (*F*_o_^2^ + 2*F*_c_^2^)/3
*S* = 1.18	(Δ/σ)_max_ = 0.001
5929 reflections	Δρ_max_ = 1.14 e Å^−^^3^
329 parameters	Δρ_min_ = −0.49 e Å^−^^3^
12 restraints	Extinction correction: none
Primary atom site location: structure-invariant direct methods	

### Fractional atomic coordinates and isotropic or equivalent isotropic displacement parameters (Å^2^)

**Table:**

	*x*	*y*	*z*	*U*_iso_*/*U*_eq_	Occ. (<1)
Cl1	0.3613 (6)	0.7404 (8)	0.1620 (5)	0.0398 (13)	0.17
Cl2	0.2520 (7)	0.6046 (7)	0.1699 (7)	0.0398 (13)	0.17
Cl3	0.3839 (7)	0.6288 (7)	0.1538 (7)	0.0398 (13)	0.17
S1	0.23386 (5)	0.27581 (5)	0.07002 (4)	0.0297 (3)	
S2	0.07625 (5)	0.35610 (5)	0.07921 (4)	0.0307 (3)	
S3	0.22138 (5)	0.41664 (5)	−0.09462 (4)	0.0308 (3)	
S4	0.03527 (5)	−0.10870 (5)	0.16513 (4)	0.0260 (2)	
P1	0.21057 (5)	0.38934 (5)	0.02920 (4)	0.0231 (2)	
P2	0.0000	0.0000	0.13345 (6)	0.0185 (3)	
C1	0.29317 (18)	0.39837 (19)	0.02368 (14)	0.0241 (8)	
C2	0.34626 (18)	0.45189 (19)	−0.00033 (15)	0.0268 (8)	
H2	0.3415	0.4884	−0.0121	0.032*	
C3	0.4062 (2)	0.4540 (2)	−0.00774 (15)	0.0302 (9)	
H3	0.4417	0.4910	−0.0255	0.036*	
C4	0.41479 (19)	0.4038 (2)	0.01014 (16)	0.0304 (9)	
H4	0.4567	0.4062	0.0055	0.036*	
C5	0.36333 (19)	0.3492 (2)	0.03509 (15)	0.0286 (9)	
H5	0.3701	0.3143	0.0480	0.034*	
C6	0.30210 (18)	0.34504 (18)	0.04128 (14)	0.0243 (8)	
C7	0.2637 (2)	0.2188 (2)	0.07750 (18)	0.0359 (10)	
H7A	0.2267	0.1749	0.0880	0.054*	
H7B	0.2985	0.2353	0.1049	0.054*	
H7C	0.2824	0.2147	0.0437	0.054*	
C8	0.20467 (18)	0.39982 (18)	0.10092 (15)	0.0233 (8)	
C9	0.2594 (2)	0.4248 (2)	0.13414 (16)	0.0347 (10)	
H9	0.3029	0.4425	0.1194	0.042*	
C10	0.2520 (2)	0.4246 (3)	0.18775 (17)	0.0403 (11)	
H10	0.2904	0.4399	0.2098	0.048*	
C11	0.1919 (3)	0.4032 (2)	0.21056 (17)	0.0413 (11)	
H11	0.1881	0.4050	0.2481	0.050*	
C12	0.1353 (2)	0.3784 (2)	0.17813 (16)	0.0316 (9)	
H12	0.0922	0.3619	0.1934	0.038*	
C13	0.14268 (19)	0.37816 (18)	0.12409 (16)	0.0257 (8)	
C14	0.0046 (2)	0.3277 (2)	0.1209 (2)	0.0464 (13)	
H14A	−0.0357	0.3098	0.0989	0.070*	
H14B	0.0087	0.3652	0.1421	0.070*	
H14C	0.0015	0.2926	0.1447	0.070*	
C15	0.22337 (17)	0.46852 (18)	0.00343 (15)	0.0243 (8)	
C16	0.22705 (18)	0.51897 (19)	0.03555 (16)	0.0279 (9)	
H16	0.2279	0.5141	0.0730	0.034*	
C17	0.2295 (2)	0.5742 (2)	0.01652 (17)	0.0376 (10)	
H17	0.2304	0.6067	0.0402	0.045*	
C18	0.2308 (2)	0.5838 (2)	−0.03719 (17)	0.0327 (9)	
H18	0.2337	0.6235	−0.0509	0.039*	
C19	0.22791 (19)	0.53560 (19)	−0.07160 (17)	0.0290 (9)	
H19	0.2290	0.5422	−0.1090	0.035*	
C20	0.22342 (17)	0.47825 (18)	−0.05158 (15)	0.0241 (8)	
C21	0.1884 (2)	0.4301 (2)	−0.15450 (15)	0.0305 (9)	
H21A	0.1774	0.3932	−0.1792	0.046*	
H21B	0.2219	0.4723	−0.1708	0.046*	
H21C	0.1480	0.4322	−0.1464	0.046*	
C22	0.06906 (16)	−0.00245 (17)	0.10086 (14)	0.0181 (7)	
C23	0.10721 (17)	0.04262 (17)	0.06167 (14)	0.0209 (7)	
H23	0.0951	0.0739	0.0491	0.025*	
C24	0.16278 (17)	0.04311 (17)	0.04031 (14)	0.0202 (7)	
H24	0.1887	0.0748	0.0137	0.024*	
C25	0.17988 (18)	−0.00197 (18)	0.05765 (14)	0.0225 (8)	
H25	0.2178	−0.0020	0.0430	0.027*	
C26	0.14256 (17)	−0.04786 (18)	0.09647 (14)	0.0223 (8)	
H26	0.1550	−0.0792	0.1081	0.027*	
C27	0.08693 (17)	−0.04882 (17)	0.11873 (14)	0.0204 (7)	
C28	0.08728 (19)	−0.13719 (19)	0.19390 (15)	0.0273 (8)	
H28A	0.0631	−0.1686	0.2228	0.041*	
H28B	0.1280	−0.0989	0.2080	0.041*	
H28C	0.0993	−0.1597	0.1667	0.041*	
C29	0.3291 (7)	0.6581 (8)	0.1414 (7)	0.032 (5)	0.17
H29	0.3225	0.6570	0.1020	0.038*	0.17

### Atomic displacement parameters (Å^2^)

**Table:**

	*U*^11^	*U*^22^	*U*^33^	*U*^12^	*U*^13^	*U*^23^
Cl1	0.055 (4)	0.037 (5)	0.028 (5)	0.024 (3)	0.006 (3)	0.003 (2)
Cl2	0.055 (4)	0.037 (5)	0.028 (5)	0.024 (3)	0.006 (3)	0.003 (2)
Cl3	0.055 (4)	0.037 (5)	0.028 (5)	0.024 (3)	0.006 (3)	0.003 (2)
S1	0.0303 (5)	0.0261 (5)	0.0302 (6)	0.0122 (4)	0.0060 (4)	0.0036 (4)
S2	0.0232 (5)	0.0291 (5)	0.0390 (6)	0.0125 (4)	−0.0012 (4)	−0.0026 (4)
S3	0.0447 (6)	0.0364 (6)	0.0198 (5)	0.0268 (5)	−0.0003 (4)	0.0004 (4)
S4	0.0226 (5)	0.0253 (5)	0.0301 (6)	0.0121 (4)	0.0043 (4)	0.0102 (4)
P1	0.0226 (5)	0.0268 (5)	0.0186 (5)	0.0114 (4)	0.0006 (4)	0.0006 (4)
P2	0.0195 (5)	0.0195 (5)	0.0166 (8)	0.0097 (2)	0.000	0.000
C1	0.0242 (19)	0.031 (2)	0.0183 (19)	0.0145 (16)	0.0002 (14)	−0.0035 (15)
C2	0.026 (2)	0.026 (2)	0.023 (2)	0.0084 (16)	−0.0042 (15)	−0.0034 (15)
C3	0.031 (2)	0.032 (2)	0.025 (2)	0.0142 (18)	0.0017 (17)	−0.0015 (17)
C4	0.0223 (19)	0.034 (2)	0.031 (2)	0.0114 (17)	−0.0014 (16)	−0.0081 (17)
C5	0.030 (2)	0.035 (2)	0.024 (2)	0.0191 (18)	0.0000 (16)	−0.0044 (16)
C6	0.028 (2)	0.0250 (19)	0.0163 (19)	0.0112 (16)	−0.0056 (15)	−0.0038 (15)
C7	0.036 (2)	0.029 (2)	0.041 (3)	0.0158 (19)	−0.0034 (19)	0.0022 (18)
C8	0.0282 (19)	0.0280 (19)	0.0192 (19)	0.0181 (17)	0.0027 (15)	0.0035 (15)
C9	0.029 (2)	0.055 (3)	0.026 (2)	0.025 (2)	−0.0013 (17)	−0.0074 (19)
C10	0.043 (3)	0.078 (3)	0.026 (2)	0.050 (3)	−0.0058 (19)	−0.003 (2)
C11	0.074 (3)	0.055 (3)	0.018 (2)	0.050 (3)	0.001 (2)	0.0003 (19)
C12	0.048 (2)	0.036 (2)	0.022 (2)	0.029 (2)	0.0137 (18)	0.0120 (17)
C13	0.031 (2)	0.0224 (19)	0.029 (2)	0.0171 (16)	0.0040 (16)	0.0039 (15)
C14	0.031 (2)	0.035 (2)	0.074 (4)	0.016 (2)	0.019 (2)	0.002 (2)
C15	0.0212 (18)	0.0258 (19)	0.027 (2)	0.0123 (16)	−0.0007 (15)	−0.0003 (15)
C16	0.030 (2)	0.029 (2)	0.020 (2)	0.0112 (17)	−0.0014 (16)	−0.0098 (16)
C17	0.043 (3)	0.033 (2)	0.033 (3)	0.017 (2)	0.000 (2)	−0.0094 (19)
C18	0.034 (2)	0.029 (2)	0.036 (2)	0.0163 (18)	−0.0002 (18)	0.0026 (18)
C19	0.030 (2)	0.033 (2)	0.025 (2)	0.0169 (18)	0.0003 (16)	0.0043 (17)
C20	0.0192 (18)	0.0257 (19)	0.027 (2)	0.0110 (15)	−0.0006 (15)	−0.0027 (16)
C21	0.035 (2)	0.038 (2)	0.022 (2)	0.0209 (19)	−0.0017 (17)	−0.0005 (17)
C22	0.0165 (16)	0.0204 (17)	0.0174 (18)	0.0092 (14)	−0.0027 (13)	−0.0026 (14)
C23	0.0232 (18)	0.0244 (18)	0.0173 (19)	0.0135 (15)	−0.0037 (14)	−0.0009 (14)
C24	0.0208 (17)	0.0188 (17)	0.0174 (18)	0.0072 (14)	0.0005 (14)	−0.0008 (14)
C25	0.0216 (18)	0.0274 (19)	0.0178 (19)	0.0117 (15)	0.0020 (14)	−0.0002 (15)
C26	0.0232 (18)	0.0216 (18)	0.023 (2)	0.0118 (15)	−0.0017 (15)	0.0010 (15)
C27	0.0187 (17)	0.0180 (17)	0.0198 (19)	0.0058 (14)	−0.0022 (14)	−0.0009 (14)
C28	0.032 (2)	0.031 (2)	0.024 (2)	0.0200 (18)	−0.0015 (16)	0.0055 (16)
C29	0.035 (8)	0.027 (9)	0.035 (7)	0.018 (7)	0.004 (10)	−0.018 (8)

### Geometric parameters (Å, °)

**Table:**

Cl1—C29	1.737 (9)	C10—H10	0.9500
Cl2—C29	1.736 (9)	C11—C12	1.398 (6)
Cl3—C29	1.736 (9)	C11—H11	0.9500
S1—C7	1.776 (4)	C12—C13	1.370 (5)
S1—C6	1.744 (4)	C12—H12	0.9500
S2—C14	1.783 (4)	C14—H14A	0.9800
S2—C13	1.762 (4)	C14—H14B	0.9800
S3—C21	1.783 (4)	C14—H14C	0.9800
S3—C20	1.769 (4)	C15—C16	1.385 (5)
S4—C27	1.745 (4)	C15—C20	1.401 (5)
S4—C28	1.786 (4)	C16—C17	1.337 (6)
P1—C1	1.817 (4)	C16—H16	0.9500
P1—C15	1.819 (4)	C17—C18	1.367 (6)
P1—C8	1.834 (4)	C17—H17	0.9500
P2—C22^i^	1.819 (3)	C18—C19	1.385 (6)
P2—C22	1.819 (3)	C18—H18	0.9500
P2—C22^ii^	1.819 (3)	C19—C20	1.371 (5)
C1—C2	1.371 (5)	C19—H19	0.9500
C1—C6	1.417 (5)	C21—H21A	0.9800
C2—C3	1.373 (5)	C21—H21B	0.9800
C2—H2	0.9500	C21—H21C	0.9800
C3—C4	1.346 (6)	C22—C23	1.383 (5)
C3—H3	0.9500	C22—C27	1.400 (5)
C4—C5	1.378 (5)	C23—C24	1.386 (5)
C4—H4	0.9500	C23—H23	0.9500
C5—C6	1.377 (5)	C24—C25	1.357 (5)
C5—H5	0.9500	C24—H24	0.9500
C7—H7A	0.9800	C25—C26	1.380 (5)
C7—H7B	0.9800	C25—H25	0.9500
C7—H7C	0.9800	C26—C27	1.391 (5)
C8—C13	1.387 (5)	C26—H26	0.9500
C8—C9	1.378 (5)	C28—H28A	0.9800
C9—C10	1.358 (6)	C28—H28B	0.9800
C9—H9	0.9500	C28—H28C	0.9800
C10—C11	1.347 (6)	C29—H29	1.0000
			
C7—S1—C6	102.45 (19)	H14A—C14—H14C	109.5
C14—S2—C13	104.0 (2)	H14B—C14—H14C	109.5
C21—S3—C20	102.65 (18)	C16—C15—C20	116.5 (3)
C27—S4—C28	104.09 (17)	C16—C15—P1	123.3 (3)
C1—P1—C15	102.93 (17)	C20—C15—P1	120.0 (3)
C1—P1—C8	101.70 (16)	C17—C16—C15	123.3 (4)
C15—P1—C8	101.72 (17)	C17—C16—H16	118.3
C22^i^—P2—C22	101.27 (14)	C15—C16—H16	118.3
C22^i^—P2—C22^ii^	101.27 (14)	C16—C17—C18	119.7 (4)
C22—P2—C22^ii^	101.27 (14)	C16—C17—H17	120.1
C2—C1—C6	117.9 (3)	C18—C17—H17	120.1
C2—C1—P1	123.4 (3)	C17—C18—C19	119.9 (4)
C6—C1—P1	118.5 (3)	C17—C18—H18	120.1
C3—C2—C1	121.5 (4)	C19—C18—H18	120.1
C3—C2—H2	119.2	C20—C19—C18	119.8 (4)
C1—C2—H2	119.2	C20—C19—H19	120.1
C4—C3—C2	120.3 (4)	C18—C19—H19	120.1
C4—C3—H3	119.9	C19—C20—C15	120.7 (4)
C2—C3—H3	119.9	C19—C20—S3	120.7 (3)
C3—C4—C5	120.6 (4)	C15—C20—S3	118.5 (3)
C3—C4—H4	119.7	S3—C21—H21A	109.5
C5—C4—H4	119.7	S3—C21—H21B	109.5
C4—C5—C6	120.0 (4)	H21A—C21—H21B	109.5
C4—C5—H5	120.0	S3—C21—H21C	109.5
C6—C5—H5	120.0	H21A—C21—H21C	109.5
C5—C6—C1	119.7 (4)	H21B—C21—H21C	109.5
C5—C6—S1	122.4 (3)	C23—C22—C27	118.9 (3)
C1—C6—S1	117.9 (3)	C23—C22—P2	122.4 (3)
S1—C7—H7A	109.5	C27—C22—P2	118.5 (3)
S1—C7—H7B	109.5	C24—C23—C22	121.4 (3)
H7A—C7—H7B	109.5	C24—C23—H23	119.3
S1—C7—H7C	109.5	C22—C23—H23	119.3
H7A—C7—H7C	109.5	C25—C24—C23	119.5 (3)
H7B—C7—H7C	109.5	C25—C24—H24	120.2
C13—C8—C9	117.5 (4)	C23—C24—H24	120.2
C13—C8—P1	119.8 (3)	C24—C25—C26	120.4 (3)
C9—C8—P1	122.6 (3)	C24—C25—H25	119.8
C10—C9—C8	120.8 (4)	C26—C25—H25	119.8
C10—C9—H9	119.6	C25—C26—C27	120.9 (3)
C8—C9—H9	119.6	C25—C26—H26	119.5
C11—C10—C9	121.9 (4)	C27—C26—H26	119.5
C11—C10—H10	119.0	C26—C27—C22	118.8 (3)
C9—C10—H10	119.0	C26—C27—S4	122.8 (3)
C10—C11—C12	118.9 (4)	C22—C27—S4	118.3 (3)
C10—C11—H11	120.5	S4—C28—H28A	109.5
C12—C11—H11	120.5	S4—C28—H28B	109.5
C11—C12—C13	119.2 (4)	H28A—C28—H28B	109.5
C11—C12—H12	120.4	S4—C28—H28C	109.5
C13—C12—H12	120.4	H28A—C28—H28C	109.5
C8—C13—C12	121.6 (4)	H28B—C28—H28C	109.5
C8—C13—S2	115.3 (3)	Cl1—C29—Cl3	112.2 (7)
C12—C13—S2	123.0 (3)	Cl1—C29—Cl2	112.4 (7)
S2—C14—H14A	109.5	Cl3—C29—Cl2	109.7 (7)
S2—C14—H14B	109.5	Cl1—C29—H29	107.5
H14A—C14—H14B	109.5	Cl3—C29—H29	107.5
S2—C14—H14C	109.5	Cl2—C29—H29	107.5
			
C15—P1—C1—C2	−6.0 (4)	C1—P1—C15—C16	−104.6 (3)
C8—P1—C1—C2	−111.1 (3)	C8—P1—C15—C16	0.5 (4)
C15—P1—C1—C6	179.1 (3)	C1—P1—C15—C20	81.7 (3)
C8—P1—C1—C6	74.0 (3)	C8—P1—C15—C20	−173.2 (3)
C6—C1—C2—C3	0.6 (6)	C20—C15—C16—C17	1.0 (6)
P1—C1—C2—C3	−174.3 (3)	P1—C15—C16—C17	−172.9 (3)
C1—C2—C3—C4	−2.1 (6)	C15—C16—C17—C18	−2.3 (6)
C2—C3—C4—C5	1.3 (6)	C16—C17—C18—C19	1.5 (6)
C3—C4—C5—C6	0.9 (6)	C17—C18—C19—C20	0.3 (6)
C4—C5—C6—C1	−2.4 (6)	C18—C19—C20—C15	−1.6 (6)
C4—C5—C6—S1	177.3 (3)	C18—C19—C20—S3	−178.7 (3)
C2—C1—C6—C5	1.6 (5)	C16—C15—C20—C19	0.9 (5)
P1—C1—C6—C5	176.7 (3)	P1—C15—C20—C19	175.0 (3)
C2—C1—C6—S1	−178.1 (3)	C16—C15—C20—S3	178.1 (3)
P1—C1—C6—S1	−3.0 (4)	P1—C15—C20—S3	−7.8 (4)
C7—S1—C6—C5	−7.1 (4)	C21—S3—C20—C19	−25.5 (4)
C7—S1—C6—C1	172.6 (3)	C21—S3—C20—C15	157.3 (3)
C1—P1—C8—C13	−161.9 (3)	C22^i^—P2—C22—C23	103.4 (2)
C15—P1—C8—C13	92.1 (3)	C22^ii^—P2—C22—C23	−0.7 (3)
C1—P1—C8—C9	13.9 (4)	C22^i^—P2—C22—C27	−81.1 (4)
C15—P1—C8—C9	−92.2 (4)	C22^ii^—P2—C22—C27	174.9 (3)
C13—C8—C9—C10	3.6 (6)	C27—C22—C23—C24	−0.6 (5)
P1—C8—C9—C10	−172.2 (3)	P2—C22—C23—C24	174.9 (3)
C8—C9—C10—C11	−3.3 (7)	C22—C23—C24—C25	0.8 (5)
C9—C10—C11—C12	2.3 (7)	C23—C24—C25—C26	−0.3 (5)
C10—C11—C12—C13	−1.7 (6)	C24—C25—C26—C27	−0.2 (5)
C9—C8—C13—C12	−3.1 (6)	C25—C26—C27—C22	0.3 (5)
P1—C8—C13—C12	172.8 (3)	C25—C26—C27—S4	176.2 (3)
C9—C8—C13—S2	172.8 (3)	C23—C22—C27—C26	0.1 (5)
P1—C8—C13—S2	−11.3 (4)	P2—C22—C27—C26	−175.6 (3)
C11—C12—C13—C8	2.2 (6)	C23—C22—C27—S4	−176.0 (3)
C11—C12—C13—S2	−173.4 (3)	P2—C22—C27—S4	8.3 (4)
C14—S2—C13—C8	177.7 (3)	C28—S4—C27—C26	23.3 (4)
C14—S2—C13—C12	−6.5 (4)	C28—S4—C27—C22	−160.7 (3)

Symmetry codes: (i) −*x*+*y*, −*x*, *z*; (ii) −*y*, *x*−*y*, *z*.
